# An Unusual Case of Pulmonary Adenocarcinoma with Multiple and Extraordinary Metastases

**DOI:** 10.5812/iranjradiol.7733

**Published:** 2012-06-30

**Authors:** Hamid Reza Haghighatkhah, Morteza Sanei Taheri, Seyed Mohammad Hadi Kharrazi, Damoon Ghazanfari Amlashi, Mehrnoosh Haddadi, Mahin Pourabdollah

**Affiliations:** 1 Department of Radiology, Shohada-e-Tajrish Hospital, Shahid Beheshti University of Medical Sciences, Tehran, Iran; 2 Department of Radiology, Shahid Beheshti University of Medical Sciences, Tehran, Iran; 3 Chronic Respiratory Diseases Research Center, National Research Institute of Tuberculosis and Lung Diseases (NRITLD), Masih Daneshvari Hospital, Shahid Beheshti University of Medical Sciences, Tehran, Iran

**Keywords:** Lung Neoplasms, Neoplasm Metastasis, Adenocarcinoma, Tomography, X-Ray Computed

## Abstract

Pulmonary adenocarcinoma is one of the major types of lung cancers in which metastasis is not uncommon. Hereby, we report a case of pulmonary adenocarcinoma with multiple muscular, cutaneous, pancreatic and peritoneal metastases. Actually, all these features occurring in one patient makes it an extraordinary case. A rare anatomic variation, double inferior vena cava (IVCs), was another rare manifestation in this case.

## 1. Introduction

Pulmonary adenocarcinoma is one of the major types of primary lung cancers accounting for approximately one third of all primary pulmonary cancers (1). Although a minority of patients with lung cancer are asymptomatic, which are usually detected in routine chest radiography, most patients present with some signs or symptoms. Metastasis is not uncommon in pulmonary neoplasms; for example, they metastasize to the adrenal glands (35% of cases), pancreas (up to 18% of cases), the skin (up to 12% of cases), CNS (up to 18% of cases) and the pleura (33% of cases) (2-14), but we have found a case with multiple extraordinary extrapulmonary metastases, which according to our knowledge, with all these features together has not been reported yet.

## 2. Case Presentation

A 37-year-old nonsmoker man with proved metastatic pulmonary adenocarcinoma came to our imaging ward to be assessed by CT scan of the chest, abdomen and pelvis about 8 months after the initial diagnosis. Findings in previous imaging investigations included left-sided pleural effusion, soft tissue density mass in the left upper pulmonary lobe, collapse/consolidation in the right upper pulmonary lobe, mediastinal and hilar lymphadenopathy, oval and round lesions with ring enhancement scattered throughout the right cerebral hemisphere and bilateral paraventricular and supraparietal regions 3-10 mm in size with surrounding edema in some areas and a small hypodensity in the right adrenal gland suggestive of metastasis or nonfunctional adenoma.

In the chest and abdominopelvic CT scan with contrast media the following abnormalities were found, some of which were really extraordinary:

Extensive collapse/consolidation in the majority of the left lung (Figure 1 A), left axillary lymphadenopathy (Figure 1 A), anterior mediastinal mass (Figure 1 A), subcutaneous nodules in the chest (Figure 1 A, B, C), bilateral moderate pleural effusion and moderate pericardial effusion with tumor adhesion and pericardial tumoral invasion (Figure 1 C), left basal segment collapse (Figure 1D), invasion to the pleura with extension to the left chest wall and chest musculature (Figure 1 A, C, E, F), patchy alveolar infiltration that could be metastatic lesions, multiple hypodense lesions in the liver suggestive of metastasis (Figure 1 E), necrotizing celiac lymphadenopathy (Figure 1 E), a large metastatic lesion in the right adrenal gland and a small lesion in the left adrenal gland (Figure 1 F), a hypodense lesion in the head of the pancreas and dilated main pancreatic duct (Figure 1 F), multiple blastic lesions in the vertebrae (Figure 1 G), multiple subcutaneous nodules in the abdominal wall, some with ring enhancement and some with nodular enhancement (nodular enhancing lesions were suggestive of subcutaneous metastatic implant and given that there were no tenderness, rubor or any other signs of abscess, the ring enhancing lesions were suggestive of metastasis as well) (Figure 1 H-J), right intrarenal metastasis (Figure 1 H) and multiple peritoneal and retroperitoneal metastatic implantations in Morison’s pouch, right subhepatic space, left perirenal space and perisplenic space (Figure 1 E, H-K).

In this case of unusual metastatic pulmonary adenocarcinoma, a rare normal variation was seen as well: double inferior vena cava (IVC) with left IVC draining to the right IVC through the left renal vein (Figures 1 F and J). Unfortunately, the patient died a few days after this last imaging.

A 37-year-old man with proved metastatic pulmonary adenocarcinoma

**Figure 1A:**
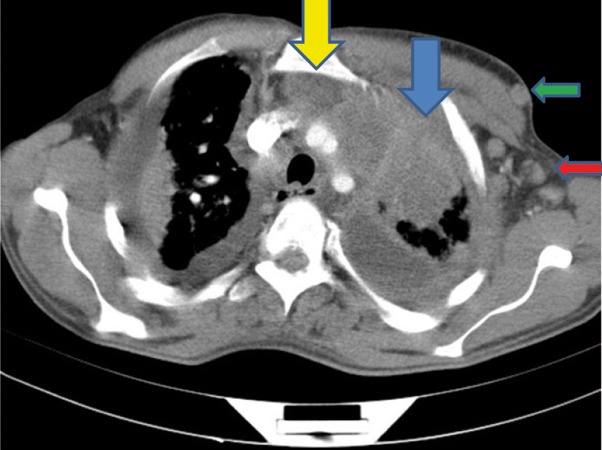
A, Contrtast-enhanced CT (CECT) of the thorax shows left axillary lymphadenopathy (red arrow), subcutaneous nodule anterior to the left pectoralis major (green arrow), left upper lobe mass with collapse/consolidation (blue arrow) and anterior mediastinal adenopathy (yellow arrow).

**Figure 1B:**
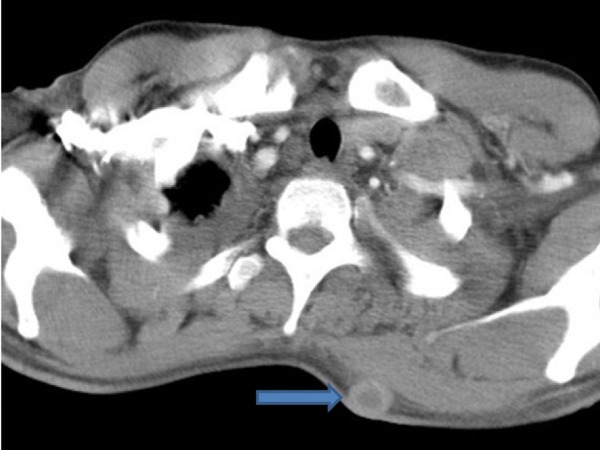
B, CECT of the upper thorax showing subcutaneous nodule in the posterior chest wall (blue arrow).

**Figure 1C:**
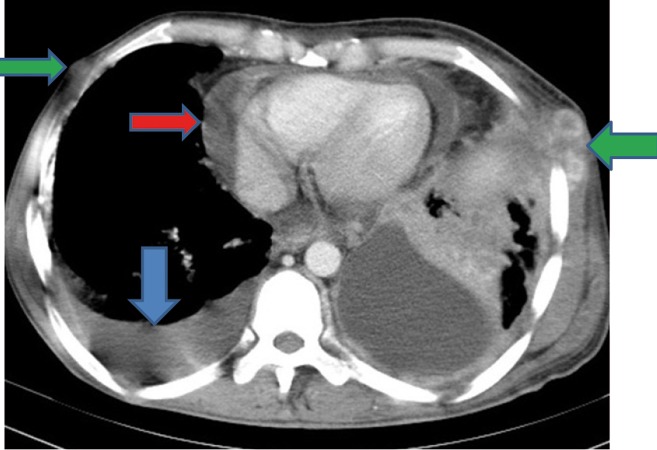
C, CECT of the thorax shows pleural effusion (blue arrow), pericardial effusion (red arrow), left intercostal muscle involvement and subcutaneous nodules (green arrows).

**Figure 1D:**
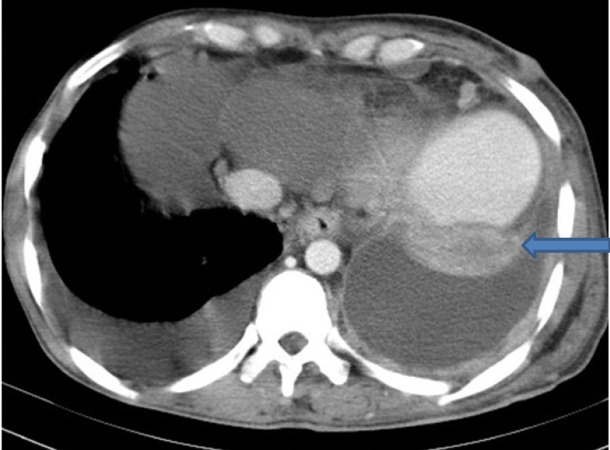
D, CECT of the lower thorax shows left basal segment collapse (blue arrow).

**Figure 1E:**
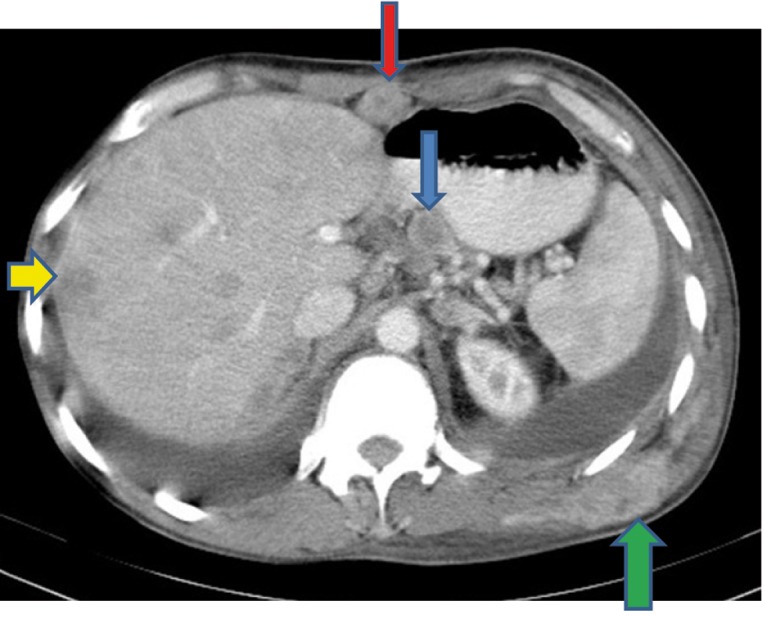
E, CECT of the upper abdomen shows necrotizing celiac lymphadenopathy (blue arrow), anterior peritoneal seeding (red arrow), liver metastases (yellow arrow) and left latissimus dorsi muscle involvement (green arrow).

**Figure 1F:**
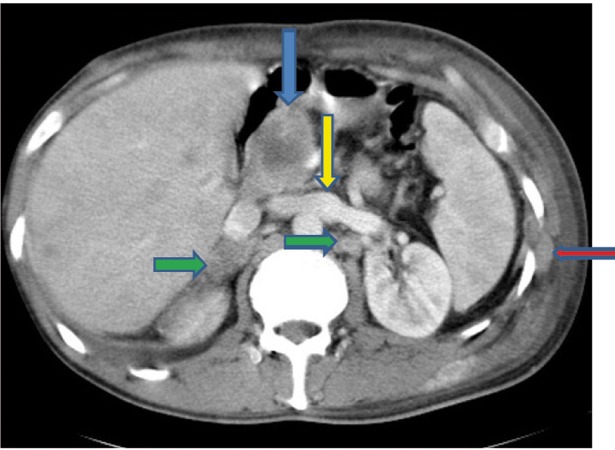
F, CECT of the abdomen shows metastasis in the head of the pancreas (blue arrow) and both adrenals (green arrows), intercostal muscle involvement (red arrow) and joining of the right and left IVCs (yellow arrow).

**Figure 1G:**
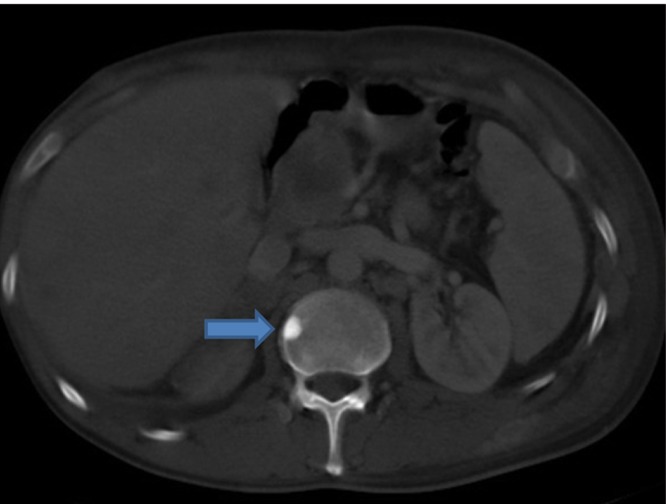
G, Abdominal CT with bone window shows sclerotic metastasis in the vertebral body (blue arrow).

**Figure 1H:**
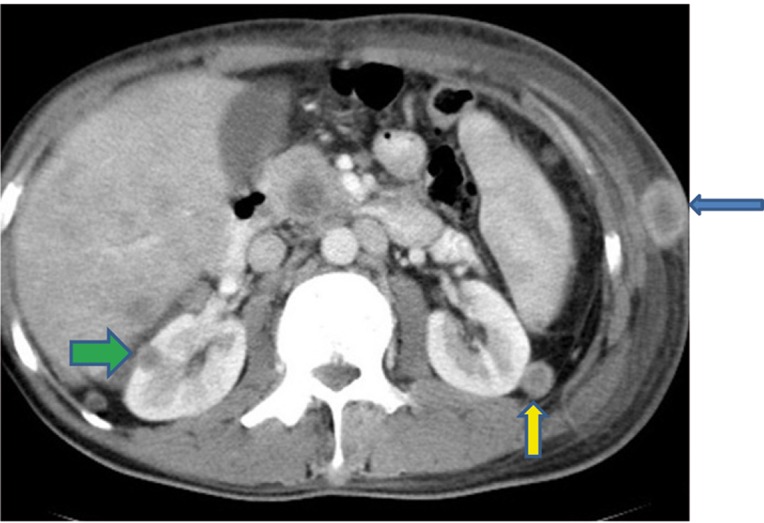
H, CECT of the abdomen shows a subcutaneous nodule (blue arrow), right intrarenal metastasis (green arrow) and left perirenal metastasis (yellow arrow).

**Figure 1I:**
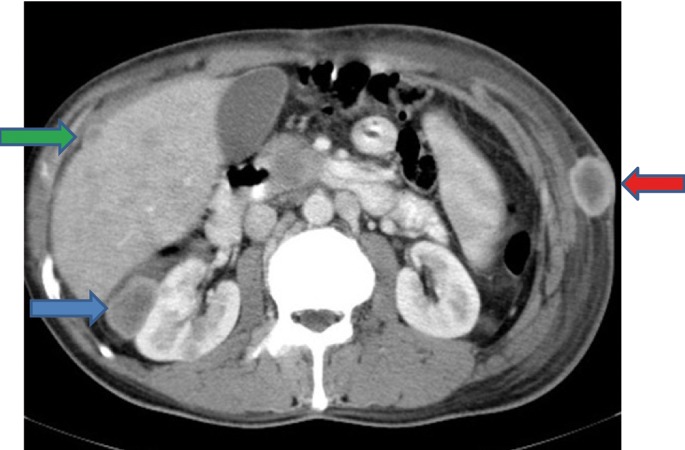
I, CECT of the abdomen shows metastasis in Morison’s pouch (blue arrow) and in the anterior peritoneal surface of the liver (g green arrow) and a subcutaneous nodule (red arrow).

**Figure 1J:**
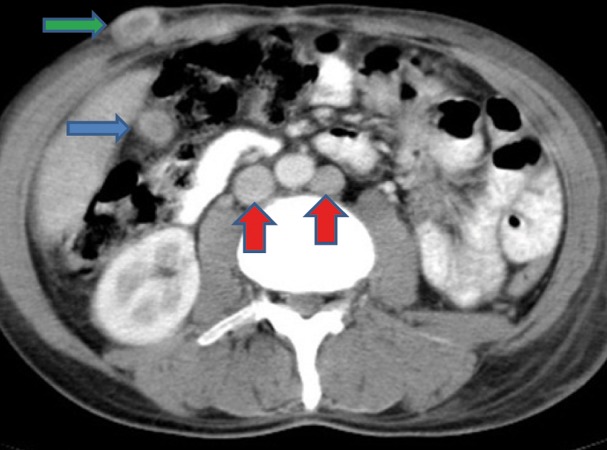
J, CECT of the abdomen shows right subhepatic space metastasis (blue arrow), a subcutaneous nodule (green arrow) and double IVCs (red arrows).

**Figure 1K:**
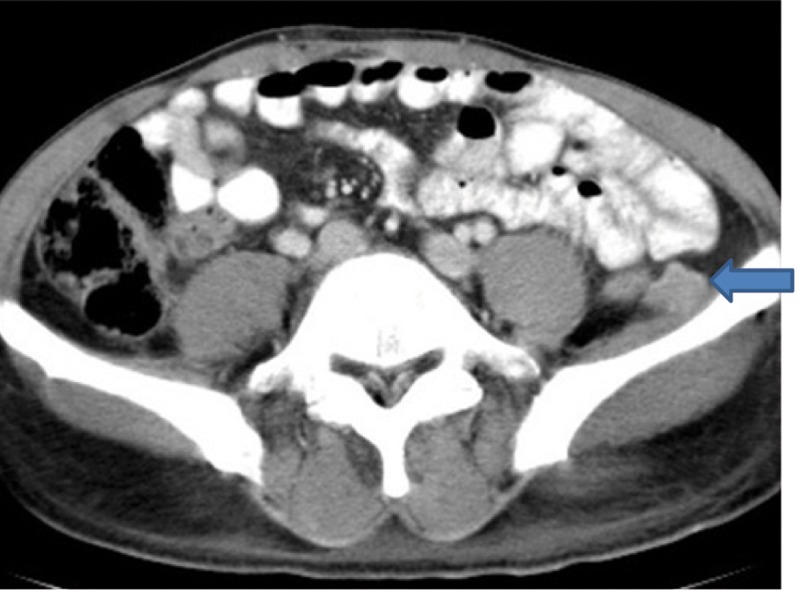
K, CECT of the pelvis shows left retroperitoneal metastasis (blue arrow).

## 3. Discussion

Lung cancers give rise to signs and symptoms caused by local tumor growth, invasion or obstruction of adjacent structures, growth in regional nodes through lymphatic spread, growth in distant metastatic sites after hematogenous dissemination and paraneoplastic syndromes.

Adenocarcinoma is the most common type of primary lung cancer (accounting for about one third of all primary lung cancers) and the most common subtype of lung cancer in nonsmokers. Lung cancer metastasis may occur in virtually every organ system. Patients with non-small cell lung cancer commonly have extrathoracic metastases to the adrenal glands, liver, brain, bones and lymph nodes at presentation. Approximately one third of patients with lung cancer will present with symptoms related to extrathoracic spread (15). Here we review some of the patient’s metastatic features in the literature. Metastases to the adrenal glands are common and are detected in up to 20% of patients at presentation (16). In view of autopsy cases, the incidence of adrenal metastasis in patients with lung cancers is about 35% (2). San Miquel et al. reported one case of bilateral adrenal metastasis from primary pulmonary adenocarcinoma (17).

The incidence of secondary pancreatic tumors has been reported in 15% of autopsy studies (18). The lung cancers infrequently metastasize to the pancreas (0-18% in different studies) (3-7). The majority of those with pancreatic metastasis are small-cell ones. In a study, among patients with small-cell lung cancer, 10.5% (13/124 cases) had pancreatic metastases, while among patients with adenocarcinoma, only 2.3% (9/379 cases) had pancreatic metastases (7).

The skin is a rare metastatic site of internal malignancies (8, 9 , 19). Breast, pulmonary, gastric and renal cancers spread to the skin more frequently than other malignancies (20, 21). In general, 0.6-12% of the patients with lung cancer develop cutaneous metastasis (8-12, 19, 22). Adenocarcinoma has the greatest tendency for skin metastasis among lung cancers (8, 10 , 22, 23). The lesions are mostly nodular and multiple (8, 22, 23). The metastasis may be located anywhere including the thorax, back, abdomen, limbs or the umbilicus (Sister Mary Joseph’s nodule) (8, 20, 21, 23-25). In a study conducted by Hidaka et al., the incidence of skin metastasis was just 2.8% among 579 cases of primary pulmonary cancers. All the cutaneous lesions were nodular and the most frequent location was the back 8. In another study, Terashima and Kanazawa reviewed 510 autopsies of lung cancers and found 13 cases of pulmonary adenocarcinoma with skin metastasis 9. Coslett and Katlic found eight cases of skin metastasis from lung cancers during a 30-month period, none of which had adenocarcinoma as the primary pulmonary cancer (20). Although skeletal muscles have an abundant blood supply, hematogenous metastatic disease to the skeletal muscle is extremely rare. Some presumptive causes include muscle motion and mechanical tumor destruction, inappropriate muscle pH for tumoral cells and the ability of the muscle to remove tumor-produced lactic acid that induces tumor neovascularity. An autopsy series suggests that muscular metastasis incidence could be as low as 0.8%. A rim-enhancing mass with central hypoattenuation has been reported as the most common appearance, occurring in 83% of lesions. Intramuscular abscesses may have a similar appearance, but clinical findings can direct the management (26, 27). Lung carcinoma seems to be the underlying primary cancer in most of these cases. Many other tumors, such as kidney, stomach, pancreas, thyroid gland, breast, ovary, prostate and bladder cancers have also been reported for secondarily spread to the muscles (28-32). Baser et al. found an enhancing mass in the right rhomboideus major muscle that was found to be metastatic from primary pulmonary adenocarcinoma (27). Lozic et al. reported a woman with non-small-cell lung cancer with nodal metastasis to the right gluteal muscle and subcutaneous tissue near the muscle (33). CNS metastases are common and are detected in up to 18% of patients with lung cancer at presentation(13). Up to 33% of patients with non-small cell lung cancer have pleural metastases at presentation(14). Metastases are 20 times more common than primary liver malignancies. Hepatic metastases most commonly originate from the GI tract, breast, and lung(34). Peritoneal seeding of malignancy occurs most commonly from ovarian cancer as well as GI tract, pancreatic and biliary cancers. Occasionally, an extraabdominal malignancy, such as breast cancer, can metastasize to the peritoneum (35), but peritoneal seeding from lung cancers has rarely been reported. Kaira and colleagues have reported a case of G-CSF producing lung cancer metastasizing to the peritoneum as well as the adrenal glands, gallbladder, intestine, pancreas, liver, and skin (36). Marta and colleagues have reported another case of pulmonary adenocarcinoma with peritoneal metastasis as well as renal, suprarenal, hepatic, bony, and lymph node metastases (37). Although metastasis to the bone is not uncommon in lung cancer, osteoblastic ones have been very rare. For example, Miyazaki et al. described a case of pulmonary adenocarcinoma and a case of pulmonary squamous-cell carcinoma with sclerotic bone metastasis, but in their cases, no other sites for metastasis were reported (38).

Finally, a rare anatomic variation, duplicated IVC, was seen in this patient. This abnormality is present in 3% of the population and is a persistence of both right and left supracardinal veins. The left IVC is the continuation of the left iliac vein and crosses the midline to join the right IVC, usually via the left renal vein (39).
